# Role of the Peripheral Nervous System in Skeletal Development and Regeneration: Controversies and Clinical Implications

**DOI:** 10.1007/s11914-023-00815-5

**Published:** 2023-08-14

**Authors:** Mohamed G. Hassan, Allison L. Horenberg, Ariella Coler-Reilly, Warren L. Grayson, Erica L. Scheller

**Affiliations:** 1 Department of Medicine, Division of Bone and Mineral Diseases, Washington University, 660 South Euclid Avenue, Campus Box 8301, St. Louis, MO 63110 USA; 2 Department of Biomedical Engineering, Johns Hopkins University, Baltimore, MD USA; 3 Translational Tissue Engineering Center, Johns Hopkins University, Baltimore, MD USA; 4 Department of Chemical and Biomolecular Engineering, Johns Hopkins University, Baltimore, MD USA; 5 Department of Materials Science and Engineering, Johns Hopkins University, Baltimore, MD USA; 6 Institute for Nanobiotechnology, Johns Hopkins University, Baltimore, MD USA; 7 Department of Biomedical Engineering, Washington University, MO St. Louis, USA; 8 Department of Cell Biology and Physiology, Washington University, MO St. Louis, USA

**Keywords:** Bone regeneration, Peripheral nerve, NGF/TrkA, Skeletal development, Denervation, Fracture healing

## Abstract

**Purpose of Review:**

This review examines the diverse functional relationships that exist between the peripheral nervous system (PNS) and bone, including key advances over the past century that inform our efforts to translate these discoveries for skeletal repair.

**Recent Findings:**

The innervation of the bone during development, homeostasis, and regeneration is highly patterned. Consistent with this, there have been nearly 100 studies over the past century that have used denervation approaches to isolate the effects of the different branches of the PNS on the bone. Overall, a common theme of balance emerges whereby an orchestration of both local and systemic neural functions must align to promote optimal skeletal repair while limiting negative consequences such as pain.

**Summary:**

An improved understanding of the functional bidirectional pathways linking the PNS and bone has important implications for skeletal development and regeneration. Clinical advances over the next century will necessitate a rigorous identification of the mechanisms underlying these effects that is cautious not to oversimplify the in vivo condition in diverse states of health and disease.

## Introduction

**Fig. 1:**
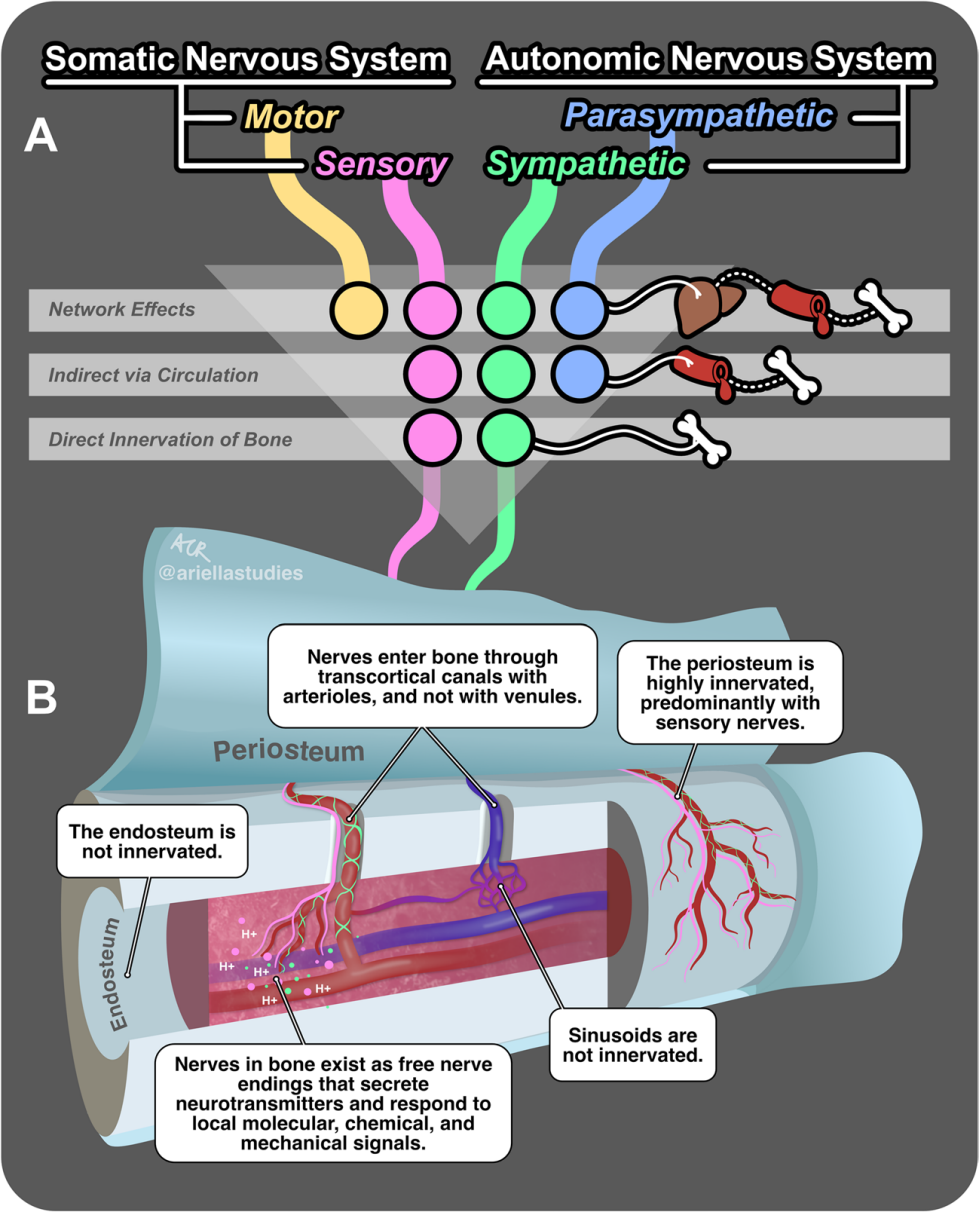
Innervation and regulation of the bone by the peripheral nervous system (PNS). (A) Three pathways of regulation of the bone by the PNS: (1) network effects, where all four divisions of the PNS indirectly influence the bone via their actions on diverse organ systems (represented by a liver icon) and downstream circulating factors (blood vessel icon); (2) indirect effects via circulation, where the sensory, parasympathetic, and sympathetic divisions of the PNS influence the bone by secreting neurotransmitters into the bloodstream; and (3) direct innervation of the bone, where only the sensory and sympathetic divisions directly release signals into the local bone microenvironment. Solid lines represent direct innervation, and dotted lines represent subsequent indirect effects mediated by circulating factors. (B) Direct innervation of bone structures by the sensory and sympathetic divisions of the PNS. The colored lines originating from (A) continue into this figure as nerve fibers from the sensory (pink) and sympathetic (green) divisions. This figure provides a deconstructed view of a bone, with the periosteum being pulled away from the surface and the bone sliced to expose the marrow and vessels within. Multiple call-out boxes elucidate the interactions of these nerve fibers with various bone structures. The colored circles near the nerve endings represent neurotransmitters secreted by these nerve fibers in response to local molecular, chemical (e.g., H +), and mechanical signals

The skeleton and the peripheral nervous system (PNS) are not independent. Instead, there is a continuous bidirectional relationship across both that facilitates optimal function [1]. This can include direct, local actions of nerves on bone cells or delivery of circulating neurotransmitters through the bloodstream (Fig. 1(A), (B)). Conversely, secreted factors from skeletal cells and biomechanical signals can modulate bone-to-brain interoceptive pathways and global peripheral nerve function. Beyond this, there are many layers of possible regulation. For example, neural signals in distant organ systems such as the gut, pancreas, or liver can regulate the bone through the modulation of circulating and humoral factors [2]. PNS function can also modify the immune response. In addition, changes in peripheral nerve function can alter muscle, behavior, and movement, resulting in local adaptation of the bone due to altered biomechanical loading [3]. Though complex, these broad potential relationships between the PNS and bone present a unique opportunity for discovery. Indeed, it has become increasingly clear that an improved understanding of neural signaling pathways has important implications for skeletal development and regeneration. In this review, we will first provide an overview of the peripheral innervation of the bone during development and repair. Next, we will comprehensively summarize the outcomes from denervation experiments performed since the 1900s that inform our understanding of the in vivo relationships between the PNS and bone. Last, we will discuss the developing clinical implications and applications for PNS pathways in skeletal regeneration.

## Innervation of the Bone from Development to Regeneration

### Coordinated Neural Infiltration Occurs During Skeletal Development

The bone contains a widely distributed neurovascular system that includes both sensory and sympathetic nerve fibers [4•, 5, 6, 7•, 8, 9••, 10••] (Fig. 1). By contrast, the bone is not directly innervated by the motor or parasympathetic divisions of the PNS (reviewed in [11]). This well-defined pattern of skeletal innervation is established during development and is highly conserved between vertebrate species [8, 12, 13]. Axons extend from the central nervous system (CNS) to peripheral targets during gestation (day 9 to day 13 in mouse) [14]. Consistent with this, in rats, the first signs of sensory innervation of the limb skeleton have been detected in the perichondrial tissue at gestational day 15 and within the bone organ at neonatal day 4 [8, 12]. This matches the infiltration of tropomyosin receptor kinase A (TrkA) expressing sensory nerves at the primary ossification centers at gestational day 14.5 in mice primarily in response to nerve growth factor (NGF) expression by the perichondrial cells [13]. Overall, nerves and blood vessels infiltrate simultaneously in areas with high osteogenic and chondrogenic activities close to the growth plate, with sensory nerves infiltrating during gestation, approximately 10 days before the postnatal recruitment of sympathetic autonomic axons [8]. This neuronal temporal delay is synchronized with the mineralization of the primary ossification centers [8, 12]. The early presence of sensory and sympathetic nerve fibers is similarly seen during intramembranous bone development in neonatal periods [15, 16].

After development, the innervation of the mature skeleton takes on several key features. First, the highest innervation density is in the periosteum, followed by the bone marrow and cortical bone [4•, 5, 6, 7•, 9••] (Fig. 1). While sensory nerves predominate in the periosteum, this is reversed to favor sympathetic axons within the bone marrow. There is also evidence of regional variation, for example, with the thoracic vertebra having higher levels of sensory innervation than the neurocranium [7•]. Second, consistent with their vasoregulatory role, most nerves in and on the bone are closely associated with the arteriolar vascular network (> 95% in the bone/marrow; ~ 80% in periosteum) [4•, 6, 11]. In humans, this means that nerve fibers are widely distributed with arterioles throughout the Haversian canal system, and in smaller species such as rodents, nerves run with nutrient arteries through transcortical canals to enter the bone marrow. By contrast, venous sinusoids in the bone and elsewhere throughout the body are not innervated. There are also many regions of the bone and bone marrow that are relatively or completely aneural. The endosteum, for example, is essentially devoid of innervation [4•, 6]. Third, there are no nerve cell bodies or true synapses in the bone. Nerve cell bodies are located in the ganglia at the level of the spinal cord (or corresponding craniofacial ganglia) [11]. Sensory and sympathetic nerves then target the bone through unipolar axonal extensions, referred to as free nerve endings because they do not synapse with other cells. Instead, they exist locally within the environment and, depending on the nerve type, relay information to the CNS based on local changes in pressure, ions, metabolites, and/or soluble factors [11, 17, 18]. Conversely, axons in bone signal to surrounding cells through the bulk release of neurotransmitters and neuropeptides. Additional information about neuronal subtypes, targeting, and function is beyond the scope of this review but has been discussed elsewhere [11, 17–20].

### The Skeletal Neural Network Undergoes Active Remodeling During Bone Regeneration and Repair

Neural infiltration following skeletal injury is a well-described phenomenon that contributes to pain [21–23] but may also be important for adequate healing. Secreted nerve recruitment factors termed neurotrophins are found throughout regions of bone regeneration with expression from diverse neural and non-neural cell types, including osteolineage cells, chondrocytes, macrophages, osteoclasts, and vascular cells [13, 24, 25, 26•, 27, 28]. Neurotrophins promote axonal growth into the injured site and, in some cases, may also act directly on local skeletal or endothelial cells to promote osteogenesis and angiogenesis, respectively [28–30]. Expression of the neurotrophin NGF, a TrkA receptor agonist, is driven by inflammatory signals such as interleukin-1 beta (IL-1β) and tumor necrosis factor-alpha (TNFα) that rapidly upregulate its local expression, which peaks approximately 3 days after acute bone injury [26•, 27, 28]. Recent studies also convincingly demonstrate that NGF binding to neural TrkA is the main stimulus for nerve ingrowth during skeletal regeneration [24, 26•, 27]. This is consistent with the expression of TrkA on ~ 80% of sensory and ~ 100% of sympathetic nerves in the bone [5]. Expression of the TrkC agonist, neurotrophin-3 (NT-3), follows a similar pattern after fracture, while the TrkB agonist, brain-derived neurotrophic factor (BDNF), remains elevated throughout healing [28]. In distraction osteogenesis, which provides a model to explore both endochondral and intramembranous ossification, NGF and TrkA are expressed highly during the distraction phase, while BDNF, NT-3, TrkB, and TrkC are upregulated during the consolidation phase. Chemoattractants and chemorepellants also direct the projections taken by the axonal growth cones, in addition to acting locally to regulate skeletal cells. This includes galanin, netrins, semaphorins, neuropilins, and ephrins, many of which have emerging roles in bone homeostasis and repair [31–34]. Many neuroregulatory factors can also directly stimulate angiogenesis, reinforcing the close coupling between nerve recruitment, vascularization, and healing [30, 35, 36].

Local nerve sprouting after injury expands the web of axons in the bone without changing the overall number of neurons in the CNS. This may have functional consequences for the interpolation of nearby signals through central relays and can also substantially modify the local release of neurotransmitters. After a fracture, sensory axons rapidly infiltrate the callus and periosteum with maximal innervation reported between days 1 and 7 after injury, prior to gradual regression during consolidation and healing [21, 27, 37–40]. Infiltration of nerves occurs even prior to blood vessels in some circumstances [27, 39]. Recruitment of sympathetic nerves after injury coincides with sensory peptidergic axons, likely due to the shared expression of TrkA and response to NGF by both axon subtypes [27, 38, 41]. In angular fractures, a unique tibial fracture model with site-specific changes in bone healing, sensory and sympathetic axon density is significantly higher along the concave side of the fracture site rather than the convex side, coinciding with regions of higher bone formation [37, 38]. This suggests that early neural (and perhaps combined neurovascular) infiltration may be vital to promote adequate bone formation. By contrast, in non-healing fractures, one study in humans found a lack of innervation, while another study identified neuroma-like structures in mice [42, 43]. Persistent innervation likely contributes to sustained pain responses [21, 22, 43]. However, it is unclear whether nerves that are generally suspected to play a positive role in early regeneration may also function to inhibit healing when they persist.

## Neural Contributions to Bone Homeostasis and Regeneration—Positive, Negative, Neutral, or All of the Above?

**Fig. 2:**
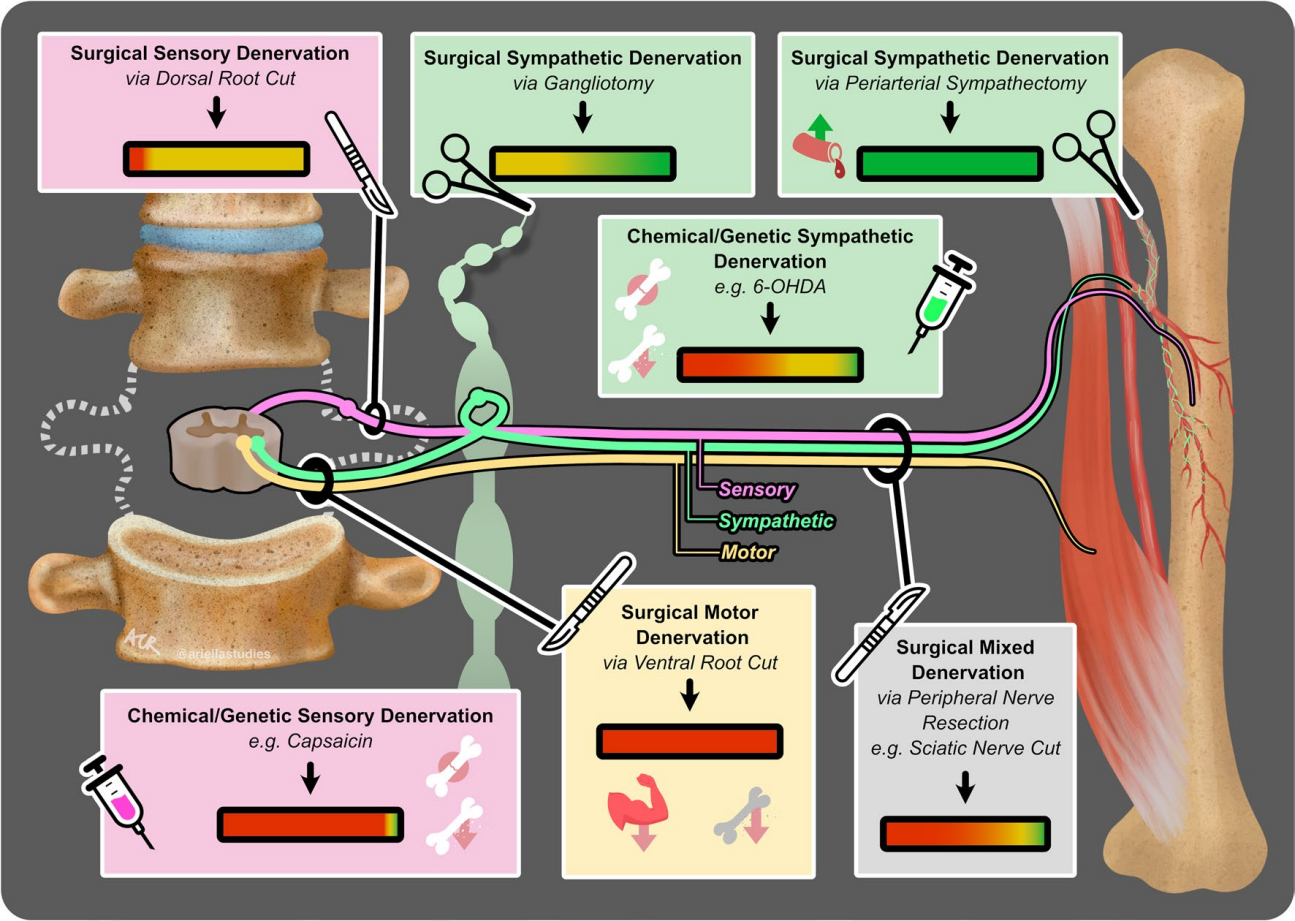
Effects of peripheral denervation on bone development, homeostasis, and repair. This figure illustrates the key methods and outcomes of 65 denervation experiments from 1900–2023, as detailed in Table 1. The schematic features a section of the spinal cord with sensory (pink), sympathetic (green), and motor (yellow) nerve fibers extending to a long bone, with accompanying muscle and vasculature. Seven boxes, each color-coded to the relevant nerve type, detail specific types of interventions and their collective outcomes. Icons within the boxes represent the method of denervation: a scalpel for surgical cuts, forceps for the removal of nervous tissue, and syringes for chemical/genetic denervation. The outcomes of these interventions are summarized by colored gradient bars (green for positive effects on the bone, yellow for neutral, and red for negative), reflecting the proportion of experimental studies yielding the indicated results. Additional icons highlight specific outcomes: artery with an upward arrow indicates increased blood flow, cracked bone signifies poor fracture healing, bone with a downward arrow represents bone loss, and muscle with a downward arrow indicates muscle loss

Since the 1800s, there have been nearly 100 studies that have used denervation approaches to isolate the effects of the different branches of the PNS on bone development, homeostasis, and repair (Fig. 2). These results are summarized below, and 65 key experiments across 51 manuscripts from 1900–2023 are presented in Table 1.

**Table 1:** Skeletal denervation studies

Study (author, year)	Model	Bone model	Denervation technique (procedure)	Bone outcome	Bone outcome summary
Targeted nerve fibers: mixed					
Xu Y et al. 2019	Mouse	Molar extraction	Surgical (unilateral inferior alveolar nerve cut)	Negative	Decreased bone formation and repair in the extraction socket. Altered immune response
Tevlin et al. 2023	Mouse	Distraction osteogenesis	Surgical (unilateral inferior alveolar nerve cut)		Decreased bone formation on the denervated side
Utagawa et al. 2023	Mouse	Homeostasis	Surgical (unilateral periosteal nerve scraping)		Decreased trabecular BV/TV and trabecular number and decreased bone formation rate
Utagawa et al. 2023	Mouse	Defect	Surgical (unilateral periosteal nerve scraping)		Decreased BV/TV and regeneration on the denervated side
Madsen et al. 1998	Rat	Fracture	Surgical (unilateral sciatic and femoral nerve cut)		Muscle atrophy after nerve cut. Increased callus size and decreased strength after denervation, even after controlling for loading with a cast
Zhang et al. 2009	Rat	Fracture	Surgical (unilateral sciatic nerve cut)		Smaller callus size and decreased healing in the denervated group
Gkiatas et al. 2019	Rat	Development	Surgical (unilateral brachial plexus cut)		20–30% decrease in bone strength on the denervated side at 6, 9, and 12 months of age
Song et al. 2012	Rabbit	Distraction osteogenesis	Surgical (unilateral sciatic nerve cut)		Decreased bone formation and slower mineralization with nerve cut, even after controlling for differences in loading
Cao et al. 2019	Rabbit	Distraction osteogenesis	Surgical (unilateral inferior alveolar nerve cut)		Lower density and formation of a new bone on the denervated side (~ − 30 to 40%)
Al-Harby et al. 1996	Dog	Fracture	Surgical (unilateral brachial plexus cut)		Impaired callus formation and failure of radiographic union on the neurectomized side
Tevlin et al. 2023	Human	Distraction osteogenesis	Injury (unilateral inferior alveolar nerve injury)		Lower BV/TV, cortical thickness, and bone formation on the side with the nerve injury
Chiego and Singh 1981	Mouse	Homeostasis	Surgical (unilateral inferior alveolar nerve cut)	Negative/neutral	Slight reductions in 3H-proline uptake by osteoblasts (− 0 to 8%), no gross change in the bone
Ma et al. 2021	Rat	Defect	Surgical (unilateral inferior alveolar nerve cut)		Decreased new bone formation on the denervated side at 1 to 4 weeks, but no change at 8 weeks post-surgery. No differences in bone mineral density at all time points
Wu et al. 2016	Rat	Homeostasis	Surgical (unilateral inferior alveolar nerve cut)		Decreased bone mass at 2 to 4 weeks after denervation, no change at 8 weeks
Dolan et al. 2022	Mouse	Digit tip regeneration	Surgical (unilateral digital nerve cut)	Neutral	Denervation did not impair digit tip regeneration if Dermabond was used to seal the amputated site. Denervation impaired soft tissue wound closure without Dermabond
Aro 1985	Rat	Fracture	Surgical (unilateral sciatic nerve cut)	Positive/neutral	Callus formation and radiographic union more rapid in denervated limb with faster formation of cartilage, but no change in callus ossification
Frymoyer and Pope 1977	Rat	Fracture	Surgical (unilateral sciatic nerve cut)	Positive	Improved fracture healing and callus strength after sciatic denervation 15–20 days following denervation and fracture
Targeted nerve fibers: sensory					
Hu et al. 2020	Mouse	Homeostasis	Chemical (30 mg/kg/day capsaicin for 1 week)	Negative	Decreased bone volume fraction and trabecular thickness in capsaicin-treated mice
Hu et al. 2020		Defect	Chemical (30 mg/kg/day capsaicin for 1 week)		Decreased new bone formation in capsaicin-treated mice
Hu et al. 2020		Fracture	Chemical (30 mg/kg/day capsaicin for 2 weeks)		Decreased new bone formation and fracture repair in capsaicin-treated mice
Heffner et al. 2014	Mouse	Development	Chemical (50 mg/kg capsaicin in neonates)		Small but significant decreases in trabecular bone structure by CT, but similar bone strength and turnover
Offley et al. 2005	Rat	Homeostasis	Chemical (25 to 50 mg/kg/day capsaicin over 2 weeks)		Decreased BMD, bone volume, and bone strength in the denervated group
Yang et al. 2022	Rat	Homeostasis	Surgical (unilateral sensory dorsal root cut)		Cancellous bone loss and increased osteoclasts
Ding et al. 2010	Rat	Development	Chemical (37.5, 75, or 150 mg/kg capsaicin)		Increased osteoclast markers and decreased strength at highest capsaicin dose
Apel et al. 2009	Rat	Fracture	Chemical (1% capsaicin along the anterior and posterior aspect of the femur, local denervation)		Larger callus cross-sectional area and decreased callus strength in the denervated group
Zhang et al. 2017	Rat	Homeostasis	Chemical (25–50 mg/kg capsaicin 3 × ; repeated every 2 weeks)		Decreased bone mass and strength
Huang et al. 2019	Rat	Implant osseointegration	Chemical (1% capsaicin along the anterior and posterior aspect of the femur, local denervation)		Decreased trabecular bone around the implant and impaired osseointegration
Zhang et al. 2017	Rat	Hindlimb suspension	Chemical (25–50 mg/kg capsaicin 3 × ; repeated every 2 weeks)	Negative/neutral	When controlling for loading with hindlimb suspension, sensory denervation with capsaicin did not reduce bone mass or strength
Grey and Carr 1915	Dog	Homeostasis	Surgical (unilateral sensory dorsal root cut)	Neutral	No change in the bone by X-ray
Eloesser 1917	Cat	Homeostasis	Surgical (unilateral sensory dorsal root cut)		Loss of reflexes, position sense, and sensation in the denervated limb. No change in the underlying strength or chemical composition of the bone, no osseous atrophy after sensory denervation. Joint disease secondary to trauma
Jimenez-Andrade et al. 2009	Rat	Fracture	Chemical (50 mg/kg capsaicin at postnatal days 1–3)	Positive/neutral	Increased callus size and no change in time to bridging/union. Reduction in pain behavior
Targeted nerve fibers: sensory + sympathetic					
Chen et al. 2019	Mouse	Homeostasis	Chemical genetic (iDTR expression in advillin-Cre + cells + diphtheria toxin)	Negative	Bone loss, decreased osteoblasts, decreased bone formation. No change in osteoclasts
Chen et al. 2019	Mouse	Homeostasis	Genetic (TrkA knockout in advillin-Cre + cells)	Negative/neutral	No change at 4 weeks. Decreased bone mass and formation at 12 weeks of age
Corbin 1937	Cat	Homeostasis	Surgical (unilateral sensory dorsal root cut + / − sympathetic gangliotomy)	Neutral	No change in the denervated bones. Trauma led to articular cartilage erosion and joint disease
Corbin 1939	Cat	Homeostasis	Surgical (unilateral sensory dorsal root cut + / − sympathetic gangliotomy)		No change in the denervated bones by X-ray, histology, or bone mass and no joint changes after limiting movement-associated trauma
Targeted nerve fibers: motor*					
Yang et al. 2022	Rat	Homeostasis	Surgical (unilateral motor ventral root cut)	Negative	Cancellous bone loss and increased osteoclasts
Grey and Carr 1915	Rabbit	Homeostasis	Surgical (unilateral motor ventral root cut)		Bone atrophy by 2 weeks after denervation with progressive bone thinning until endpoint
Targeted nerve fibers: sympathetic					
Shi et al. 2021	Mouse	Homeostasis	Chemical (6-OHDA mediated sympathetic denervation)	Negative	Significant decreases in cortical and cancellous bone mass (− 70%) and loss of bone strength
Shi et al. 2021	Mouse	Fracture	Chemical (6-OHDA mediated sympathetic denervation)		Impaired bone formation and callus strength
Wagner et al. 2022	Mouse	Defect	Chemical (6-OHDA mediated sympathetic denervation)		Decreased regeneration and bone formation after 6-OHDA sympathectomy
Scammel 1994	Rabbit	Fracture	Chemical (6-OHDA mediated sympathetic denervation)		Increased callus size but reduced callus strength in sympathectomized rabbits
Niedermair et al. 2020	Mouse	Fracture	Chemical (6-OHDA mediated sympathetic denervation in OVX mice)	Neutral	No change in cartilage and bone formation during callus repair
Hill et al. 1991	Rat	Homeostasis	Chemical (50 mg/kg/day guanethidine for 3 weeks, 1 week after birth)		No change in bone mass or bone formation rate in the tibia, but increased osteoclast recruitment after extraction
Kizilay et al. 2020	Rat	Fracture	Surgical (unilateral stellate sympathetic gangliotomy)		No difference in bone formation, union, and remodeling between groups
Harris and Mcdonald 1936	Kitten	Development	Surgical (unilateral lumbar sympathetic gangliotomy)		No change in bone growth or length after sympathectomy
Key and Moore 1933	Cat	Fracture & defect	Surgical (unilateral thoracic sympathetic gangliotomy)		No difference in fracture or defect healing between the sympathectomized or intact sides
Corbin 1939	Cat	Homeostasis	Surgical (unilateral or bilateral lumbar sympathetic gangliotomy)		No change in the bone by X-ray, histology, or bone mass
Harris and Mcdonald 1936	Puppy	Development	Surgical (unilateral lumbar sympathetic gangliotomy)		No change in bone growth or length after sympathectomy
Pearse and Morton 1930	Dog	Fracture	Surgical (unilateral lumbar sympathetic gangliotomy)		Little difference in healing with sympathectomy
Harris and Mcdonald 1936	Lamb	Development	Surgical (unilateral lumbar sympathetic gangliotomy)		No change in bone growth or length after sympathectomy
Guan et al. 2023	Rat	Homeostasis	Chemical (guanethidine-mediated sympathetic denervation)	Negative/neutral	No change in the bone in the spine, decreased trabecular BMD, BV/TV, and Tb.Th. in the tibia (~ − 30%)
Cherruau et al. 1999	Rat	Molar extraction	Chemical (40 mg/kg/day guanethidine for 21 days)	Positive	Decreased bone resorption surface and osteoclast formation
Wang 2012	Rat	Distraction osteogenesis	Surgical (unilateral cervical sympathetic gangliotomy)		Increased bone formation at 1 (+ 45%) and 14 days (+ 13%) of consolidation
Du et al. 2014	Rat	Distraction osteogenesis	Surgical (unilateral cervical sympathetic gangliotomy)		Improved bone marrow mesenchymal stem cell migration into the defect
Palma 1925, as reported in Colp 1933	Rabbit	Fracture	Surgical (unilateral cervical sympathetic gangliotomy)		Improved early vascularization and fracture healing on the sympathectomized side
Uffreduzzi 1924, as reported in Colp 1933	Rabbit	Fracture	Surgical (unilateral periarterial sympathectomy)**		Improved early vascularization and fracture healing on the sympathectomized side
Ito and Asami 1932	Dog	Fracture	Surgical (unilateral lumbar sympathetic gangliotomy)		Increased healing with lumbar sympathectomy (11/12)
Zollinger 1933	Dog	Fracture	Surgical (unilateral lumbar sympathetic gangliotomy)		Greater regeneration of the sympathectomized side in 15/17 cases but considered “slight” and not to warrant clinical application
Ito and Asami 1932	Dog	Fracture	Surgical (unilateral periarterial sympathectomy)**		Increased healing with periarterial sympathectomy (11/13)
Fontaine 1926, as reported in Colp 1933	Dog	Fracture	Surgical (unilateral periarterial sympathectomy)**		Improved callus strength and healing on the sympathectomized size
Colp 1933	Dog	Fracture	Surgical (unilateral periarterial sympathectomy)**		Improved vascularization and fracture healing on the sympathectomized size
Harris and Mcdonald 1936	Human	Development	Surgical (unilateral lumbar sympathetic gangliotomy)		Positive (acceleration or maintenance of the rate of growth of the child’s leg on the sympathectomized side in 63% of cases, no improvement in 37%)

^*^Motor nerves do not innervate the bone directly but can influence the surrounding muscle. **Periarterial sympathectomy denervates the adventitia of the main vascular branch to the bone but may not denervate the bone itself

### Mixed Denervation Approaches Reinforce the Link Between the Muscle and Bone and Provide New Clues About Neural Coordination of Soft and Hard Tissue Healing

Sir D’Arcy Thompson in 1917 said, “Between muscle and bone there can be no change in the one but it is correlated with changes in the other” [44]. The same remains true within the context of the neural regulation of the bone. Specifically, any reduction of the motor innervation of muscle will subsequently lead to progressive skeletal atrophy due to loss of muscle mass. This is most clearly shown after the transection of the ventral motor roots of the spinal cord to induce selective motor denervation of the limb [45, 46•] and has since been repeated many times in studies of peripheral nerve resection. This most commonly includes the brachial plexus (upper limb), sciatic and femoral nerves (lower limb), and inferior alveolar nerve (mandible) that contain mixed populations of motor, sensory, and sympathetic axons [47–59]. In 12/14 studies in Table 1, when mixed surgical denervation was paired with models of bone injury, denervation had a negative impact on soft tissue closure, fracture repair, or osseous defect healing.

Mixed peripheral nerve function has also been studied within the context of axolotl limb regeneration. Axolotls are capable of full limb re-growth when amputation is performed proximal to the elbow joint. Local expression of neuregulin-1 and its receptor, ErbB2, are decreased with limb denervation [60]. In addition, denervation delays regeneration, while supplementation of neuregulin-1 rescues regeneration in denervated limbs. The closest analog to this in mammals is the regeneration of the terminal portion of the digit tip. Sensory and sympathetic nerves are found in the digit tip prior to and during regeneration [61••]. However, despite work suggesting that nerves are required for complete renewal [62], a recent study found that nerves are exclusively required for soft tissue wound closure rather than bone regeneration. Thus, when open wounds were treated with Dermabond to stimulate closure, regenerated digits with denervation were morphologically similar to controls [63]. Overall, mixed denervation approaches demonstrate that an intact PNS supports optimal regeneration when present. In addition to maintaining muscle mass, this work also hints at mechanisms that may include the neural regulation of both hard and soft tissue healing.

### Studies of Surgical, but Not Chemical, Denervation Show that Depletion of Sympathetic Nerves May Promote Bone Accrual and Repair by Increasing Blood Flow

In the early 1900s, an extensive series of experiments were undertaken to understand the impact of surgical sympathetic denervation on bone development and fracture healing (Fig. 2, Table 1). The rationale was the finding that sympathetic denervation could promote local vascularization and blood flow. The first surgical method consisted of the removal of the sympathetic ganglia (e.g., unilateral lumbar gangliotomy to denervate the lower limb). This results in permanent and selective removal of the sympathetic nerves in a small body region. Fracture or bone defect healing with sympathetic gangliotomy showed either no change [64–68] or increased healing on the sympathectomized side [67, 69–71]. Unilateral lumbar sympathectomy was also performed in 46 children with leg paralysis and unilateral shortening due to poliomyelitis, to increase the growth of the affected limb [72]. Despite failed experiments in kittens, puppies, and lambs that showed no difference in limb growth, there was an acceleration or maintenance of the rate of growth of the paralyzed leg on the sympathectomized side in 63% of cases. The second approach, termed periarterial sympathectomy, consisted of the removal of the sympathetic nerve axon-containing adventitia from the vessel wall (e.g., the femoral artery for the lower limb). This causes sympathetic denervation of the vessel itself and any downstream site that was originally targeted by this vascular network. Overall, periarterial sympathectomy resulted in increased blood flow with more rapid callus formation, ossification, and healing on the sympathectomized side in dogs, rabbits, and humans [71, 73, 74]. However, despite some apparent clinical success, these procedures received substantial pushback from other members of the medical community that did not find them to be advantageous, and surgical sympathectomy has since been discontinued for growth- or fracture-related outcomes.

In contrast to the generally positive results of surgical sympathectomy, chemical sympathectomy often leads to bone loss, impaired bone strength, and impaired bone healing (Fig. 2, Table 1) [75–81]. The discrepancy between the surgical and chemical denervation models may be due to the whole-body suppression of sympathetic adrenergic systems by chemical treatment, resulting in substantial global alterations in mouse health that are not present after regional surgical denervation.

### Sensory Nerves are Required to Ensure Optimal Skeletal Loading and May Augment Bone Formation and Repair in Settings of Development and Injury

Surgical studies have also been performed to selectively disrupt the sensory innervation of the bone (Table 1). Interpretation is challenging because sensory denervation causes improper limb use and joint trauma due to altered position sense. However, when isolated from changes in biomechanics and loading, surgical sensory denervation generally does not alter bone mass or strength over long periods of time (up to 3 years in one study) [45, 46•, 66, 82–84]. In the 1930s, this led to the conclusion that skeletal sensory innervation is not necessary for the maintenance of bone. However, more recently, sensory nerves have gained renewed attention based on their extensive pattern of infiltration following bone injury. Though some studies have shown neutral effects, chemical and genetic denervation studies have generally found that local sensory denervation during bone healing impairs implant osseointegration, alters fracture callus size, and decreases bone repair [23, 85–87]. Whole-body chemical and genetic methods of sensory denervation also lead to generalized bone loss in the absence of injury [85, 88–91]. As with chemical sympathetic denervation, differences between surgical and genetic/chemical approaches may be due to the impact of global sensory denervation on peripheral systems (sight, smell, gait, etc.), in addition to any local effects. In addition, any benefits of sensory nerve recruitment for fracture repair must be balanced with clinical needs. Management of fractures is challenging, particularly in patients with multiple co-morbidities, limited mobility, and pain. Currently, local sensory neurolysis is a clinically approved adjunct to manage pain for inoperable hip fractures [92•, 93]. Neurolysis substantially improves mobility and quality of life, which can also independently promote positive clinical outcomes for these patients.

## Clinical Adaptation of Peripheral Neural Pathways for Skeletal Regeneration

The PNS mediates a multitude of critical functions throughout the body, and denervation studies over the last century show that gross modification to the PNS is generally not warranted to promote bone repair. However, the isolation of novel molecular mechanisms linking the PNS and bone has also led to the identification of several high-yield pathways that inform targeted strategies to support skeletal regeneration.

### Methods to Enhance Nerve Infiltration Are Associated with Increased Bone Healing

With sensory denervation often resulting in impaired bone healing after injury (Table 1), therapeutic studies have aimed to promote neural ingrowth and, by proxy, encourage bone formation. Nerves and vessels infiltrate within the first week following a fracture. Thus, therapeutic methods aim to improve neurovascular infiltration early in the healing process. One study utilized MMP-degradable tissue-engineered periosteum (MMP-TEP)-coated allografts to improve scaffold integration with the native tissue since periosteum can help direct neurovascular infiltration [94]. The MMP-TEP allograft group demonstrated early-stage neurovascularization and improved both mineralization and mechanical properties of femoral defects as compared to hydrogel-TEP allografts and allografts alone. An additional group of studies has aimed to encourage bone formation by redirecting the entire nerve bundles to the injury region to improve neural infiltration and neuropeptide release during healing. Positioning the cut end of the peripheral nerve trunk into tissue-engineered bone grafts (TEBGs) improved callus and bone formation compared to TEBG-only samples [95–98]. In addition, nerve bundle TEBGs increased the expression of neuropeptides and their receptors and improved vascularization in the defect region. The mechanisms involved in this model were not explored in depth; however, the implanted nerve bundles may either be supporting innervation/neuropeptide production or perhaps serving as a reservoir of pro-regenerative signals from nerve-associated cells.

### Sensory Neurotransmitter Calcitonin Gene–Related Peptide (CGRP) Can Enhance Bone Repair

One possible osteoanabolic factor is the sensory neurotransmitter CGRP. In addition to being a potent vasodilator, in vivo and in vitro studies report that CGRP promotes osteoblast differentiation and inhibits bone resorption [99]. CGRP receptor deletion in osteoprogenitors during fracture healing decreases callus and cartilage area as well as cell proliferation, resulting in an overall impairment to fracture healing [100]. Conversely, methods that increase the concentration of CGRP within skeletal defects can enhance the rate of bone formation and repair [99, 101]. For example, atypical femoral fractures, which exhibit delayed healing and can occur following bisphosphonate treatment, demonstrate lower CGRP expression, reduced bony bridging, and increased fibrous tissue formation. Local injection of CGRP (100 nM) for 14 days after injury helped to restore healing in these fractures [102••].

While exogenous neuropeptide delivery can enhance regeneration, high levels are often required. To avoid this, other therapeutic approaches aim to stimulate endogenous release by exploiting materials or systems that encourage neuronal signaling or neuropeptide expression. For example, biomaterials containing divalent metal cations such as magnesium induce local CGRP release and stimulate robust periosteum-derived stem cell osteogenic differentiation [102••, 103, 104••]. Direct stimulation of sensory nerve cell bodies in the dorsal root ganglia with an implanted microelectrical stimulation system (IMESS) also enhances CGRP production and drives spinal fusion only in IMESS-targeted areas. Similarly, in osteoporotic femoral fracture healing, IMESS at the dorsal root ganglia (20 min/day for 2 weeks) improves vascularization and fracture healing in a CGRP-dependent manner without increasing pain-like responses [105••]. Conversely, the intraperitoneal injection of CGRP inhibitors in rats negatively affected fracture healing, demonstrating a significant decrease in the formation of the mineralized callus [106]. Balancing the osteoanabolic effects of sensory neurotransmitters such as CGRP with pain outcomes is crucial since these factors can also enhance nociception [107]. Adding to the complexity, CGRP inhibitors such as atogepant and erenumab are used clinically for the treatment of migraine [108]. Development of pro-regenerative paradigms will require careful consideration of current therapeutics and the actions of target neurotransmitters across systems.

### Schwann Cell–Secreted Factors Signal Bone-Forming Cells to Activate Repair Responses

Peripheral nerve axons are wrapped by a protective sheath of myelinating or non-myelinating Schwann cells. After an injury, certain populations of Schwann cells can disassociate from damaged axons to expand at the site of injury. Schwann cells primarily modulate bone regeneration through secreted factors that signal bone-forming cells. In mouse digit tip regeneration, denervation inhibited Schwann cell infiltration, depleting Schwann cell–secreted factors, including OSM and PDGF-AA, and inhibiting regeneration [109]. Transplantation of additional Schwann cells or local injection of OSM and PDGF-AA was able to rescue the impaired healing [109]. Schwann cell–derived exosomes were also shown to enhance osteogenic differentiation in vitro and improve bone formation in vivo when included in a titanium alloy scaffold [110]. Similarly, when Schwann cells were included in scaffolds along with osteoblasts and endothelial cells, angiogenesis and vascularization were enhanced in vivo [111]. Mechanistically, in vitro studies suggest that Schwann cell–derived factors increase the proliferation of skeletal stem cells and endothelial cells while signaling through the VEGF, ERK/MAPK, and PI3k-Akt pathways [112].

### β-Blockers Increase BMD, Reduce Fracture Risk, and Promote Healing

Surgical denervation studies pinpoint the sympathetic nervous system as a negative regulator of bone (Fig. 2, Table 1). Consistent with this, norepinephrine, the primary neurotransmitter of sympathetic adrenergic axons, can act on skeletal β-adrenergic receptors to suppress bone formation and increase osteoclast function, leading to decreased bone mass [113–115]. This informs studies on the effects of β-blockers, a group of common antihypertensive medications, on bone homeostasis and repair. β-blockers inhibit the diverse effects of the neural- and adrenal-derived catecholamines, including norepinephrine. Clinical use of β-blockers is associated with reduced risk of fracture and increased bone mineral density [116–118]. Treatment with β-blockers in mouse models increases bone mass due to enhanced bone formation and decreased bone resorption [114]. The β-blocker propranolol can also promote mineral apposition, callus formation, and strength in rodent femoral defects [119]. Though it is unclear if the benefits to the bone are due to direct actions on bone cells (vs. vasoregulatory or other effects), β-blockers remain a promising therapeutic strategy for managing osteoporosis and enhancing bone healing. In addition, recent studies have identified alternate pathways that converge on the regulation of sympathetic tone to modulate bone mass. For example, genetic downregulation of PGE2 signaling by advillin-expressing sympathetic and sensory nerves or introducing divalent cations such as magnesium can suppress sympathetic activity, promoting osteoblast formation and increases in the bone [85, 91, 104••, 120]. Increased sympathetic tone has also been proposed as a putative mechanism underlying bone loss and fragility in diseases including chronic heart failure [78] and impaired fracture repair with the use of medications such as SSRIs [121], providing targeted opportunities for future intervention.

### Activation of NGF/TrkA Signaling Can Augment Bone Healing but Needs to be Balanced with Pain and Tumor-Promoting Responses

Therapeutic approaches using growth factors are popular for targeting known regeneration pathways, as they involve simple strategies to stimulate the host microenvironment. Consistent with this, local application of the neurotrophin NGF activates neuronal signaling, vascularization, and other bone resident cells that stimulate bone formation [30, 41, 122–126]. Genetic targeting approaches have shown that the bone anabolic effects are largely mediated by the activation of TrkA, the high-affinity receptor of NGF [26•, 27], with the potential for additional pro-regenerative actions of the low-affinity NGF receptor p75-NTR [127]. Specifically, when paired with rodent models of long bone fracture or calvarial defect, global inhibition of TrkA signaling reduces vascularization, osteoblastic activity, and ossification rate [26•, 27]. Conversely, treatment with synthetic TrkA agonist gambogic amide can promote angiogenesis and bone repair [128]. The mechanism underlying this effect remains to be clarified and may involve the activation of TrkA on neural, vascular, and/or local skeletal cells [30, 128]. Beyond this, targeted inhibition of p75-NTR in osteoblast precursors can restrict osteoprogenitor migration into the repair site [127]. Other nerve regulatory factors and neuropeptides such as BDNF, substance P, Sema3A, vasoactive intestinal peptide, and galanin have also been used to trigger bone healing and demonstrated similar results [70, 129–136].

NGF/TrkA signaling is also a major regulator of peripheral pain. Given this, clinical anti-NGF therapies to treat musculoskeletal pain are currently under development, and inhibition of NGF/TrkA signaling to treat fracture pain has been tested in rodents. In this case, anti-NGF and anti-TrkA antibodies successfully reduced pain behaviors without affecting fracture repair [137, 138]. In addition, NGF may also be relevant to tumorigenesis [139]. Many studies highlight the increase of NGF secretion and its receptors in the microenvironment of different cancer types [140]. For this reason, increasing attention is directed toward NGF and/or TrkA as a therapeutic target for effectively controlling tumor progression. Most recently, this includes the clinical use of an emerging class of TRK inhibitors to treat TRK fusion–positive cancers [141]. While TRK inhibitors have favorable overall safety, off-target adverse events, including weight gain, dizziness/ataxia, paraesthesias, and bone fracture, are occasionally observed [141, 142]. Future studies will be essential to understand the impact of the clinical modulation of TRK signaling on the bone microenvironment during the maintenance and healing phases.

## Conclusions

Studies on the relationships between the PNS and bone initially peaked in the early 1900s. Over 100 years later, we have now uncovered diverse links between the PNS and the skeleton that occur during bone homeostasis, development, and repair. Overall, a common theme of balance emerges whereby an orchestration of both local and systemic neural functions must align to promote optimal repair while limiting negative consequences such as pain. Advances over the next century will necessitate a rigorous identification of the mechanisms underlying these effects that is cautious not to oversimplify the in vivo condition. Clinical use of sensory neurolysis, CGRP inhibitors, and TRK-targeting therapies will undoubtedly inform our understanding of their necessity for bone health and the potential to leverage the anabolic components of these pathways to promote regeneration. In addition, β-blockers and PNS-targeting bioactive implant materials represent emerging strategies to support repair.
